# Laboratory investigation of GO-SA-MWCNTs ternary hybrid nanoparticles efficacy on dynamic viscosity and wear properties of oil (5W30) and modeling based on machine learning

**DOI:** 10.1038/s41598-023-37623-x

**Published:** 2023-06-29

**Authors:** Mojtaba Sepehrnia, Somayeh Davoodabadi Farahani, Abolfazl Hamidi Arani, Ali Taghavi, Hamidreza Golmohammadi

**Affiliations:** 1 Department of Mechanical Engineering, Technical and Vocational University, Qom, Iran; 2 Department of Mechanical Engineering, Shahabdanesh University, Qom, Iran; 3 School of Mechanical Engineering, Arak University of Technology, Arak, Iran; 4 Department of Biomedical Engineering, Shahabdanesh University, Qom, Iran

**Keywords:** Engineering, Mechanical engineering

## Abstract

In the present study, the properties of ternary hybrid nanofluid (THNF) of oil (5W30) - Graphene Oxide (GO)-Silica Aerogel (SA)-multi-walled carbon nanotubes (MWCNTs) in volume fractions ($\varphi )$ of 0.3%, 0.6%, 0.9%, 1.2%, and 1.5% and at temperatures 5 to 65 °C has been measured. This THNF is made in a two-step method and a viscometer device made in USA is used for viscosity measurements. The wear test was performed via a pin-on-disk tool according to the ASTM G99 standard. The outcomes show that the viscosity increases with the increase in the $\varphi$, and the reduction in temperature. By enhancing the temperature by 60 °C, at $\varphi$ = 1.2% and a shear rate (SR) of 50 rpm, a viscosity reduction of approximately 92% has been observed. Also, the results showed that with the rise in SR, the shear stress increased and the viscosity decreased. The estimated values of THNF viscosity at various SRs and temperatures show that its behavior is non-Newtonian. The efficacy of adding nanopowders (NPs) on the stability of the friction and wear behavior of the base oil has been studied. The findings of the test display that the wear rate and friction coefficient increased about 68% and 4.5% for $\varphi$ = 1.5% compared to $\varphi$ = 0. Neural network (NN), Adaptive Neuro-Fuzzy Inference System (ANFIS), and Gaussian process regression (GPR) based on machine learning (ML) have been used to model viscosity. Each model predicted the viscosity of the THNF well, and Rsquare > 0.99.

## Introduction

Nanofluid (NF) is a mixture that is obtained by adding nanoscale (nanometer) particles to a base fluid with the aim of improving heat transfer (HT). Nanopowders (NPs) are usually metal particles such as Ni^1^, Cu^2^, Ag^3^, AlN^4^, CaCO_3_^5^, metals oxide such as CuO^6^, SiO_2_^7^, Fe_2_O_3_^8^, Fe_3_O_4_^9^, BaTiO_3_^10^, TiO_2_^11^, Al_2_O_3_^12^, carbon compounds such as SiC^13^, MWCNTs^14^, Graphene^15^, and Graphite^16^. The primary fluid is usually water^17^, ethylene glycol^18^, propylene glycol^19^, and oil^20^. Based on analytical^21^, numerical^22^ and laboratory studies^23^, researchers showed that NFs are superior to base fluids^24^. This behavior depends on features such as the shape of NPs, their size distribution, volume fraction ($\varphi$), temperature (*T*), thermal conductivity coefficient of NPs and base fluid^25^.

During the last decade, the HT features of NFs have been extensively investigated^26^. The amount of viscosity in the design of NF is very vital and important for fluid flow. In applied projects in the industry, fluid viscosity is important due to the pressure drop and power required for pumping. In the past years, several analyses have been directed in the rheological manners of NFs in the *T* of 5 °C to 70 °C^27^. Esfe^28^ explored the efficacy of *T* and $\varphi$ on the viscosity ($\mu$) of MWCNTs–Al_2_O_3_ (3:7)/EG fluid. Also, their outcomes displayed that the relative $\mu$ increased non-linearly with the rise in $\varphi$.

Binary hybrid nanofluids (BHNF) are actually created by the dispersion of two NPs in a base fluid. M. Asadi and A. Asadi^29^ in a laboratory study estimated the $\mu$ of ZnO-MWCNTs-oil in the *T* of 5 to 55 °C and $\varphi$ of 0.125 to 1%. Given their results, the $\mu$ of NF decrements with growing *T*. Augmenting $\varphi$ makes a maximum 45% enhancement in the $\mu$ of the NF compared to the $\mu$ of the BF. Afrand et al.^30^ explored the $\mu$ of ethylene glycol-silver-iron oxide BHNF in $\varphi$ of 0.0375 to 1.2% and *T* of 25 to 50 °C. Given their findings, this BHNF has a non-Newtonian behavior (NNB) for $\varphi$ > 6%, and the $\mu$ of the BHNF lessens with mounting *T*. Bahrami et al.^31^ assessed the $\mu$ of H_2_O-EG-iron-copper oxide BHNF in $\varphi$ of 0.5 to 1.5% and *T* of 25 to 50 °C. They described that this BHNF has an NNB. The $\mu$ of this BHNF reduces with enhancing *T* and rises with rising $\varphi$. Sepehrnia et al.^32^ experimentally investigated the rheological properties of CuO-CeO_2_-10W40 BHNF over the range $\varphi$ of 0.25 to 1.5%, at *T* of 5 to 55 °C and the SRs of 20 to 1000 rpm. Findings discovered that 10W40 engine oil and BHNF exhibited NNB. Also, in another study, Sepehrnia et al.^33^ examined the rheological performance of SnO_2_-CeO_2_/SAE50 BHNF over the range $\varphi$ of 0.25–1.5%, at *T* of 25–67 °C and the SRs of 1333–2932.6 s^–1^. They observed a non-Newtonian-pseudoplastic behavior for the SAE50 and BHNF in all states. Sepehrnia et al.^34^ studied the rheological properties of the SiO_2_-MWCNTs/5W30 BHNF at different *T* of 5–65 °C, as well as $\varphi$ of 0.05–1%, and SRs of 50 to 1000 rpm. They proposed a three-variable correlation to predict relative viscosity of BHNF. Also, they developed a GMDH-NN model for predicting the relative viscosity. They proved SiO_2_-MWCNTs/5W30 BHNF has non-Newtonian-pseudoplastic behavior.

Ternary hybrid nanofluids (THNF) are produced by the dispersion of three NPs in a base fluid. Shaoo^35^ tested the hydrodynamic manner of THNF with Al_2_O_3_ (spherical), CNT (cylindrical), and Graphene (platelet). He discovered the NP shape has considerable efficacy on hydrodynamic performance. The $\mu$ of water-Al_2_O_3_-CuO-TiO_2_ THNF has been examined by shoo and Kumar^36^. They found that the increase in viscosity of THNF is about 10-50% more than BHNF. Xuan et al.^37^ determined an optimal ratio for combining NPs (Cu, CuO and Al_2_O_3_) in water on the viscosity and thermal features, which can be effective for use in thermal equipment. Said et al.^38^ synthesized the THNF (rGO-Fe_3_O_4_-TiO_2-_EG). They found that the $\mu$ augmented by 64.5% at $\varphi$ = 0.25 and *T*= 25 °C. The rheological and tribological performance of MoO_3_-MWCNTs-GO-SAE 5W30 THNF is examined by Sepehrnia et al.^39^. Their results reveal that 5W30 engine oil and THNF had a non-Newtonian-pseudoplastic behavior. In another research, Sepehrnia et al.^40^ experimentally investigated the rheological performance of SA‑GO‑CeO_2_-10W40 THNF over the range φ of 0.25 to 1.5%, at *T* of 5 to 55 °C and the SRs of 40 to 1000 rpm. The non-Newtonian-pseudoplastic behavior of THNF at all *T* and *φ* is exposed by examining power-law model coefficients.

Wear is the gradual reduction of material from the surface of an object that has been in contact with another object when these two objects move relative to each other. Unlike properties such as elasticity, wear is not an inherent feature of the material, but is related to the tribological parameters of the system. Tribo is a system that influences the wear behavior and friction of materials in contact with each other. Speed, surface quality, time, type of material and lubricant, force, temperature, surface conditions, friction, and wear are the parameters of a tribometer. The addition of NPs to psychoanalyses affects the wear rate. In this field, researchers have conducted studies. For example, Lee et al.^41^ explored the efficacy of $\mathbf{\varphi }$ on the lubrication properties of fullerene NPs combined with mineral oil. In this study, lubrication features were assessed by measuring friction surface *T* and friction coefficient (FC). The results of the tests showed that nano-oils with a larger $\boldsymbol{\varphi }$ have a smaller FC and fewer wear, which is a sign of improving the lubrication properties of mineral oil due to the addition of NPs. Wu et al.^42^ showed that adding diamond NPs to SAE30 LB51163-11 oil reduces the FC, while adding it to SAE30 LB51153 oil increases the FC. The effect of adding disulfide NPs: Molybdenum, tungsten disulfide and boron nitride with sizes of 50 to 100 nm are experimentally tested on the efficacy of standard lubricants used in forming processes. Friction and wear were two parameters investigated by Mosleh et al.^43^. Their findings show that the addition of NPs meaningfully lessens wear and friction. Hu et al.^44^ reported that the physical layer of WS2 NPs can properly hold the workpiece during the friction process that leads to wear. These NPs can be easily absorbed on the surface and leave good lubrication effects on the surface. Krishna et al.^45^ explored the efficacy of 50 nm boric acid NPs inside the base lubricants, including SAE40 oil and coconut oil with various weight percentages, as well as the surface roughness in the Turning process of AISI1040 steel. They disclosed that if nano-lubricants are used, the temperature and wear, as well as the surface roughness, are significantly reduced compared to the base lubricant. There are other explorations in this field that have obtained similar results^46^. In tribological research, artificial intelligence can open new horizons and improve understanding of wear and friction phenomena. Hasan and Karabacak^47^ used the Neural Network (ANN), Support Vector Machine (SVM), and Gaussian process regression (GPR) to predict firction coefficient. Mahakur et al.^48^ utilized the SVM to estimate the tribological behavior of epoxy composites. Lifar et al.^49^ used the ML to determine a relationship between coating hardness and experimental deposition settings. The success and high ability of artificial intelligence techniques in tribological research have been demonstrated innumerous studies^50^.

In the present study, three nanomaterials with different shapes are used, each of which has unique properties. SA, GO and MWCNTs are spherical, sheet and cylindrical shapes, respectively. MWCNTs have excellent thermal and mechanical properties. Although MWCNTs have hydrophobic properties^51^, their combination with oxide materials makes a favorable dispersion^52^, so in this regard GO is used in this research. Utilizing GO in a base fluid has been examined in previous studies^53^ and it has been confirmed that thermal properties increases. High porosity and low thermal conductivity are the properties of SA^54^. Although the low thermal conductivity of SA is one of the weaknesses of this material, its high porosity encourages researchers to use it in the production of THNFs^40^ because high porosity makes stable and homogeneous THNFs.

As mentioned in the literature review, the properties of THNFs are superior to mono NFs and BHNFs. On the other hand, very few studies have been done on THNFs, and it is necessary to investigate suitable compounds based on the properties of NPs. Therefore, based on the desirable properties of three types of GO, MWCNTs and SA, the combination of these three types of NPs along with 5W30 oil has been selected as a THNF for the present study. On the other hand, no study has been done on this compound so far. Therefore, in the current exploration, the rheological properties of the GO-SA-MWCNTs-5W30 THNF has been reviewed, and the influence of SR, *T*, and $\varphi$ on the $\mu$ have been inspected. Also, tribological performance has been done on the base oil and THNF with maximum volume fraction. Using soft computing models, $\mu$ has been estimated. These models: NN, ANFIS and GPR are based on ML and artificial intelligence.

## Experiments

The first stage in conducting the current research is the preparation of NF and the steady suspension of NPs in the primary fluid. In this research, a two-stage technique is utilized to make NF. Having stable and homogeneous samples is the most significant state for experiments with minimal lines. Clumping or deficiency of suitable suspension of NPs in the primary fluid can make a large error in the measurement. There are various ways to avoid this phenomenon. For this purpose, the NF has been rotated for 1 hour via a magnetic stirrer. The name of the stirrer is UP400S and it is made in Heishler, Germany. The weight of this device is 2.3 kg with a single-phase voltage of 230 volts and a frequency of 50 to 60 Hz and a maximum current consumption of 4 A. The NF was exposed to ultrasound waves for 2 hours by a device with a power of 230 W & a frequency of 24 kHz. To assess the $\mu$, a CAP2000+ viscometer made by Brookfield America was employed with an accuracy of ±2. The $\mu$ of NF has been measured in $\varphi$ of 0, 0.3, 0.6, 0.9, 1.2, and 1.5% for the 5 $\le T\le$ 65 °C. First, the viscometer is standardized with oil at surrounding *T*. Tests have been done for the 50 ≤ SR ≤ 950 rpm. All experiments have been repeated at each $\varphi$ and specified temperature at various SRs. The characteristics of NPS: GO, SA and MWCNTs and the characteristics of 5W30 oil are given in Tables 1 and 2, respectively.

**Table 1:** The nano-powders properties.

	GO	SA	MWCNTs
NP purity (%)	> 99.3%	99.5%	95%
Shape	2-D sheet	Spherical	Cylindrical
Color	Black	White	Black
Size	t = 0.43–1.23 nm; D=1.5–5.5μm	20 nm	Length: 50 μm
			OD: 5–15 nm
			ID: 3–5 nm
Density (ρ)	1.5 g/cm^3^	0.07 g/cm^3^	2.1 g/cm^3^
SSA	500–1200 m^2^/g	682 m^2^/g	223 m^2^/g

**Table 2:** The base fluid properties.

Kinematic viscosity @ 100 °C	cSt (11.62)
Viscosity index (VI)	161
FLASH point	220 (°C)
Pour point	− 39 (°C)
Density @ 15 °C	0.854 (g/cm^3^)

In this study, three NPs: GO, SA NPs and MWCNTs have been selected for investigation. According to Eq. (1), the $\varphi$ in terms of percentage is computed as follow as^55^:

1 $$\varphi = \frac{{\sum\limits_{i = 1}^{3} {\Psi_{np,i} } }}{{\sum\limits_{i = 1}^{3} {\Psi_{np,i} } + \Psi_{fluid} }} \times 100,$$

$\Psi$ is obtained by dividing the mass by the density of each component. *np* is related to NPs.

**Figure 1:**
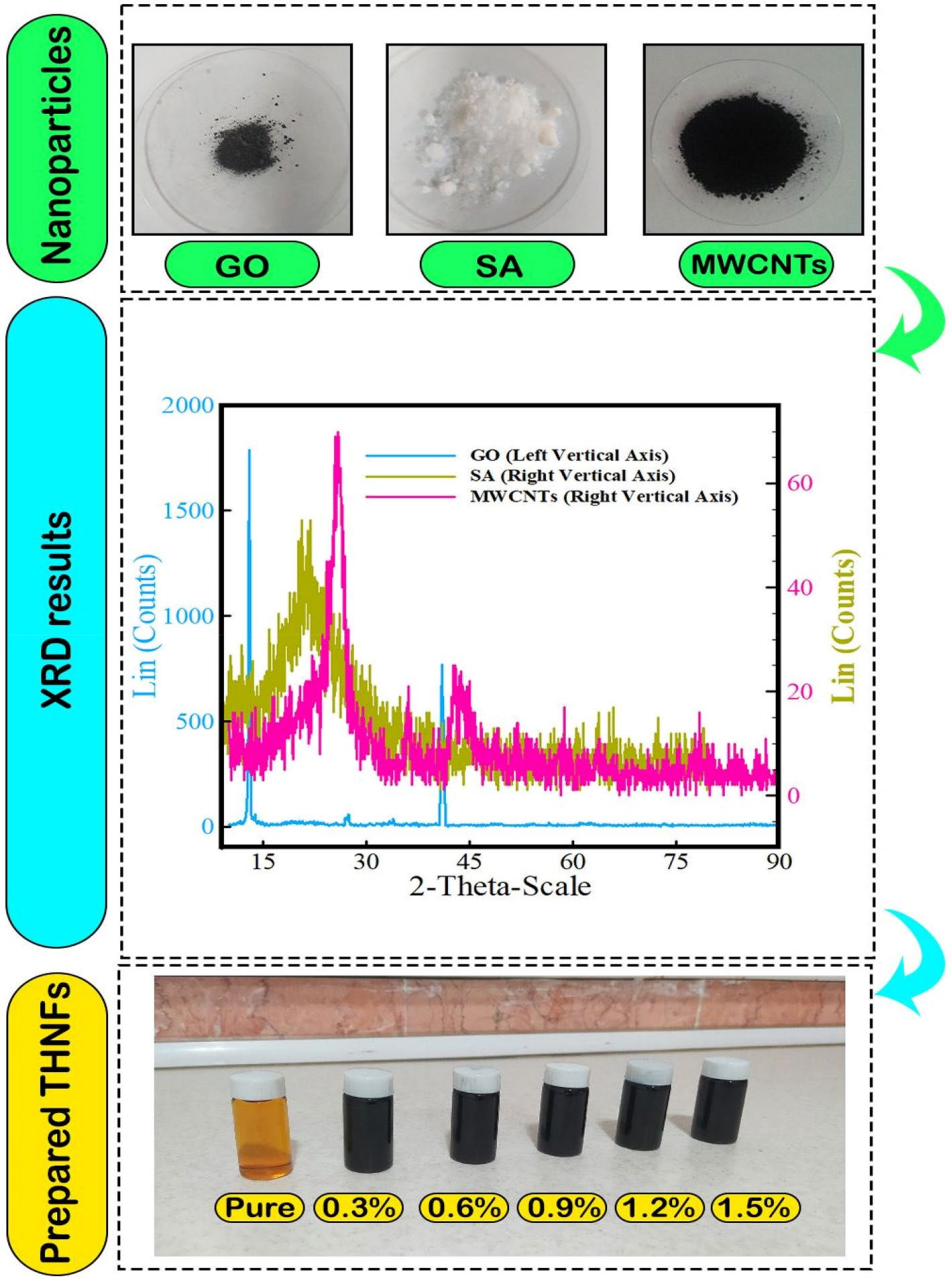
The process of the preparing three NPs, performing XRD analysis, and preparing THNFs.

X-Ray diffraction is a multi-purpose non-destructive technique that gives comprehensive info on the chemical configuration and crystal construction of natural and industrial materials. Each crystal structure has its unique X-ray diffraction form that can be utilized as a fingerprint to detect its character. In the following figures, NPs (GO, SA and MWCNTs) have their own wavelength. This analysis shows the general nature of the substance and a fingerprint for each substance. In Fig. 1, NPs used in base oil has been analyzed similarly. Results specify that three NPs have a very good crystal phase structure. The prepared THNF samples are shown in Fig. 1 in $\varphi$ of (0–1.5%), respectively. The prepared NFs were steady for 4 weeks and no lumpiness was detected.

The wear test device is a device that is used to predict the tribological behavior of engineering materials and alloys in operational conditions. This laboratory equipment calculates and stores the wear and friction coefficient of the sample due to the rotational contact between the pin and the sample in dry, fluid and high-temperature environments. A TSN-WTC-02 pin-on-disk tool base on the ASTM G99 standard was used to estimate the amount of wear. The pin and disk are made from CK45 steel and E52100 steel, respectively. A tribometer is a device for measuring friction on a surface. This device is used for testing, and simulating wear and friction, and it can also work for solid friction without lubrication and for boundary lubrication with liquid lubricant. The FC when the system is motionless is specified by enhancing the hanging weight until the object placed on the surface moves from the following equation^43^.

2 $${\text{F}} = \mu {\text{N}}.$$

Where N is the normal load, F is the loading force equivalent to the weight. In most tribometers, wear is revealed by measuring the mass of the target component before and after the test. The disc must be cleaned well with alcohol and detergent before and after each test, because by measuring the weight of the disc before and after each test, the amount of weight loss shows the amount of wear. The pin or needle located on the disc is connected to the holding arm and an electrical sensor to measure the friction force, to display the data. The controllable variables of this device are vertical force, distance and time, speed and rotation. This tribometer has software that allows this set of programs to fully customize the user for the wear test and data analysis and generate subsequent reports. During this friction measurement test and after traveling the distance, the test will stop automatically. In the wear test, the aim is to regulate the finest $\varphi$ to lessen the friction. After every stage of the FC test, the disc’s mass was computed with a precise scale with an accurateness of 0.0001 gr. Measurement of the mass was done to estimate the wear of the elements. The amount of wear can be considered^42^.

3 $$wear\, rate\left(\frac{{m}^{2}}{N}\right)=\frac{\Delta m}{\mathrm{\rho lF}},$$

where $\Delta m,\rho ,l and F$ are mass loss, density of sample, distance and the applied load.

## Results of rheological tests

### Rheological behavior

**Figure 2:**
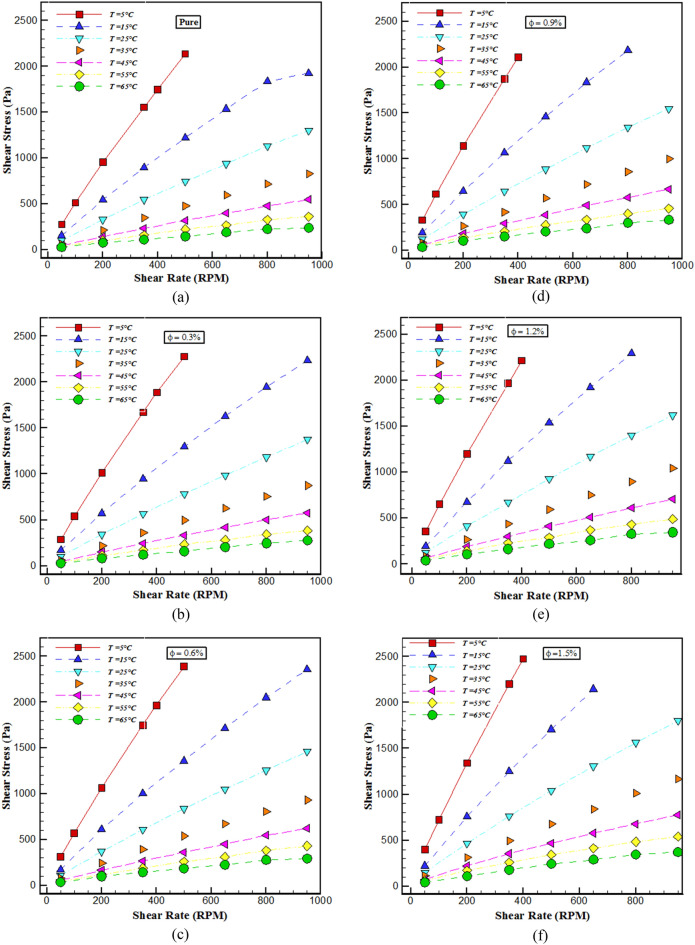
Changes in shear stress with SR.

**Figure 3:**
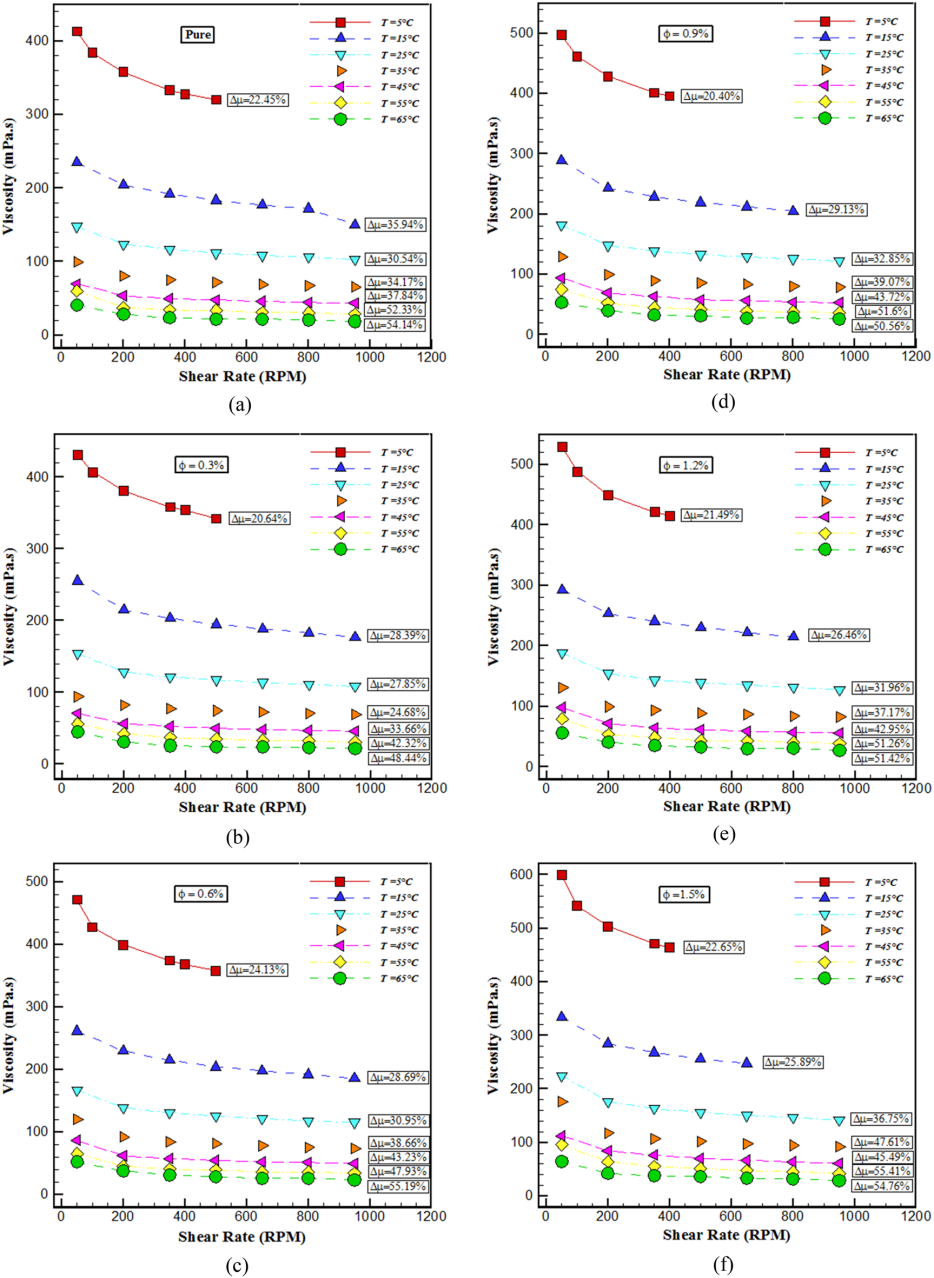
Changes in viscosity according to SR.

In this segment, several tests have been done in the field of identifying the rheological behavior of the mentioned THNF. Figure 2 displays the changes in shear stress (SS) with SR for $\varphi$ and different temperatures. In all cases, the shear stress is augmented with the increment of the SR. With mounting *T*, the SS has decreased. The cause for this behavior is the reduction of dynamic viscosity. Also, the distribution of SS with SR is non-linear, which specifies the non-Newtonian nature of the BF. Figure 3 displays the changes in $\mu$ according to SR at different temperatures. The SR factor is used to reveal the Newtonian or non-Newtonian behavior of the fluid. From the outcomes of these graphs, with the rise of the SR, the $\mu$ has decreased significantly and the fluid can flow easily. When the SR increases, the attractive force between NPs-fluid decreases and the particles disperses, which leads to a reduction in $\mu$. Owing to the non-linearity of viscosity changes with SR, it is possible to obtain a NF with NNB. Fluid viscosity at 65°C with increasing SR from 50 to 950 rpm for $\varphi$ of 0, 0.3, 0.6, 0.9, 1.2 and 1.5% reduces about 54%, 51.33%, 55.19%, 50.57%, 51.43%, and 54.77% respectively.

**Figure 4:**
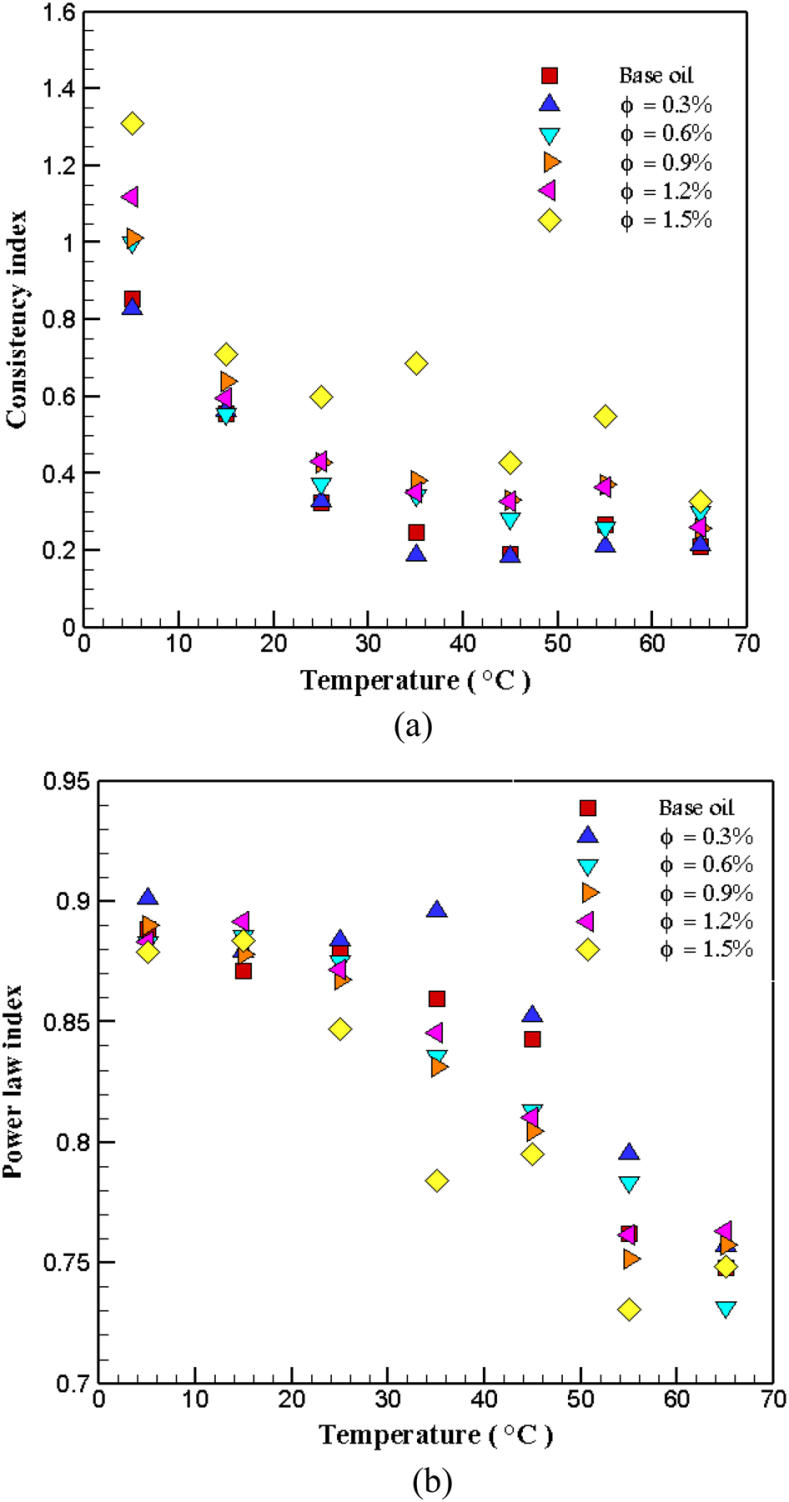
Variations of (**a**) consistency index and (**b**) power base index on temperature for different $\varphi$.

As the results showed, the variations of SS with SR are non-linear. Therefore, shear stress follows Power law. Using experimental data, the equation of power and the fit of the value of the base index of power and consistency have been obtained. Figure 4 shows the changes in the base index of power and consistency according to temperature in different $\varphi$. The values of the power base index at all *T* and $\varphi$ are less than unity, which indicates the NNB (quasi-plastic) of the prepared NF. As the temperature increases, the consistency index decreases. The $\mu$ is due to the intermolecular force (IMF), and the movement of molecules boosts with enhancing *T* and the IMF weakens. Sepehrnia et al.^39,40^ in their researches investigated THNF and approved that GO-MoO_3_-MWCNT-5W30 and GO-CeO_2_‑SA-10W40 had a non-Newtonian-pseudoplastic behavior.

### Temperature effects on the $\upmu$

**Figure 5:**
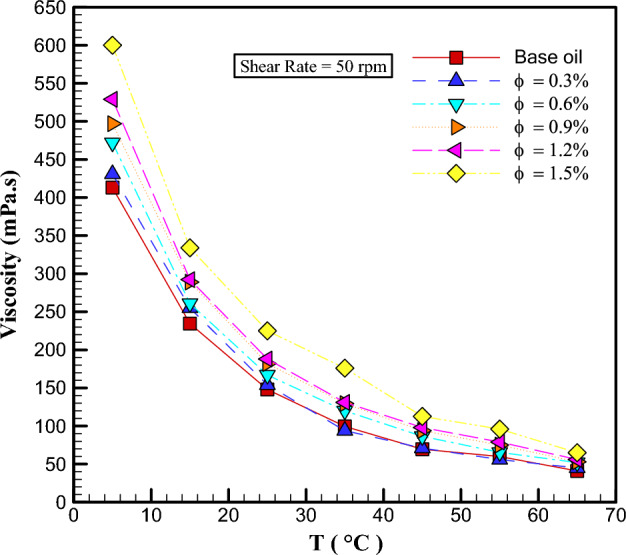
Changes in viscosity with temperature.

Figure 5 displays the changes in $\mu$ with *T* in various $\varphi$. As the *T* increases in different $\varphi$, the $\mu$ values decrease and experience a sharp drop. The rate of viscosity lessens owing to temperature augment depending on the molecular structure of the fluid. When there are NPs in the fluid, this rise leads to an enhancement in heat in the fluid and a weakening of inter-molecular forces, which ultimately makes a decrement in $\mu$. For instance, the $\mu$ of the fluid at a SR of 50 rpm has decreased by augmenting the *T* from 5 to 65 °C for $\varphi$ of 0, 0.3, 0.6, 0.9, 1.2 and 1.5%, respectively, about 90.073%, 91.89%, 88.983%, 89.336%, 89.414%, and 89.167%. The qualitative study shown in Fig. 5 agrees previous researches. For instance, Sepehrnia et al.^52^ in their research stated that the $\mu$ reduces with enhancing *T*.

### Impact of $\mathrm{\varphi }$ on $\upmu$

**Figure 6:**
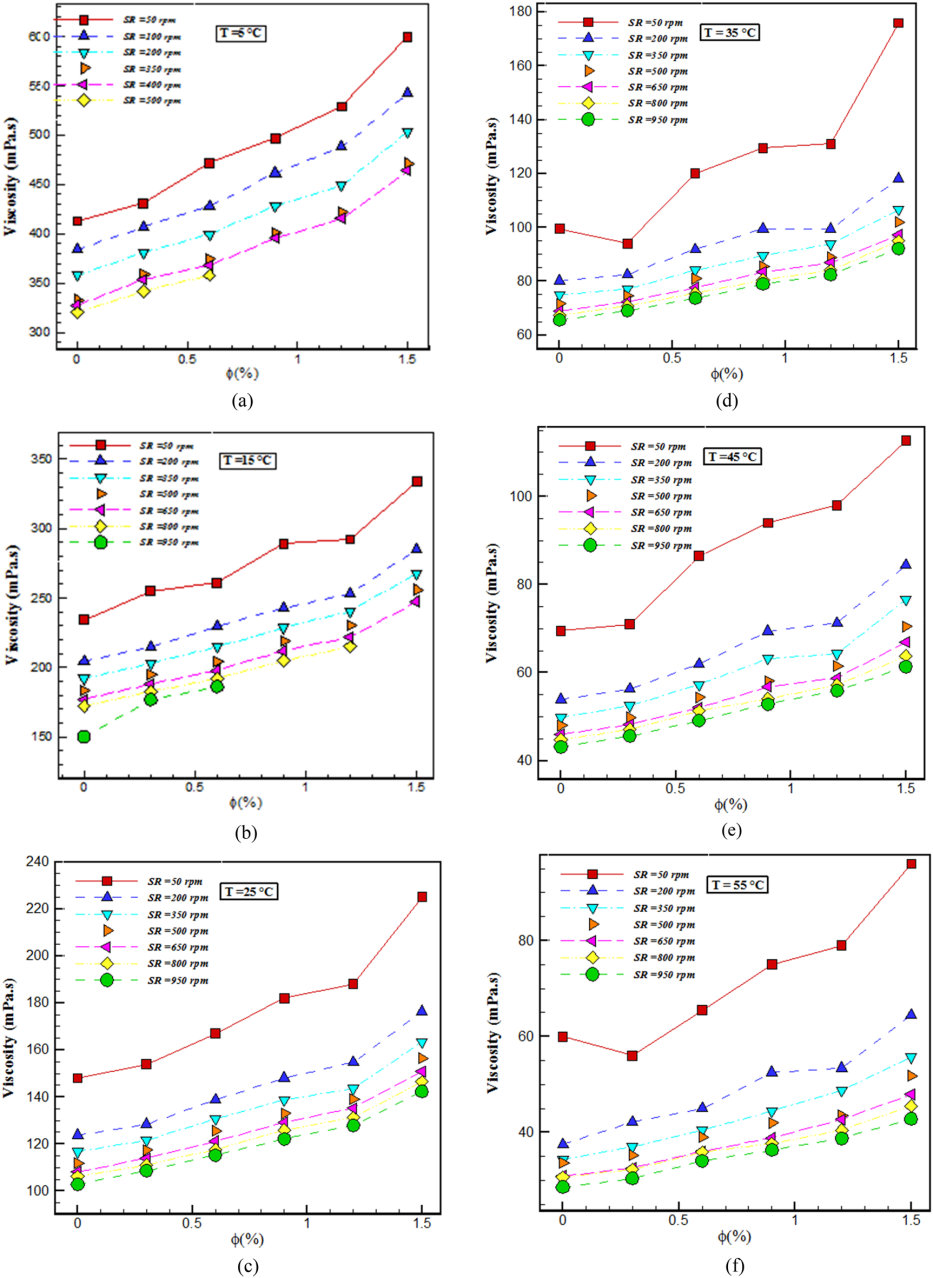

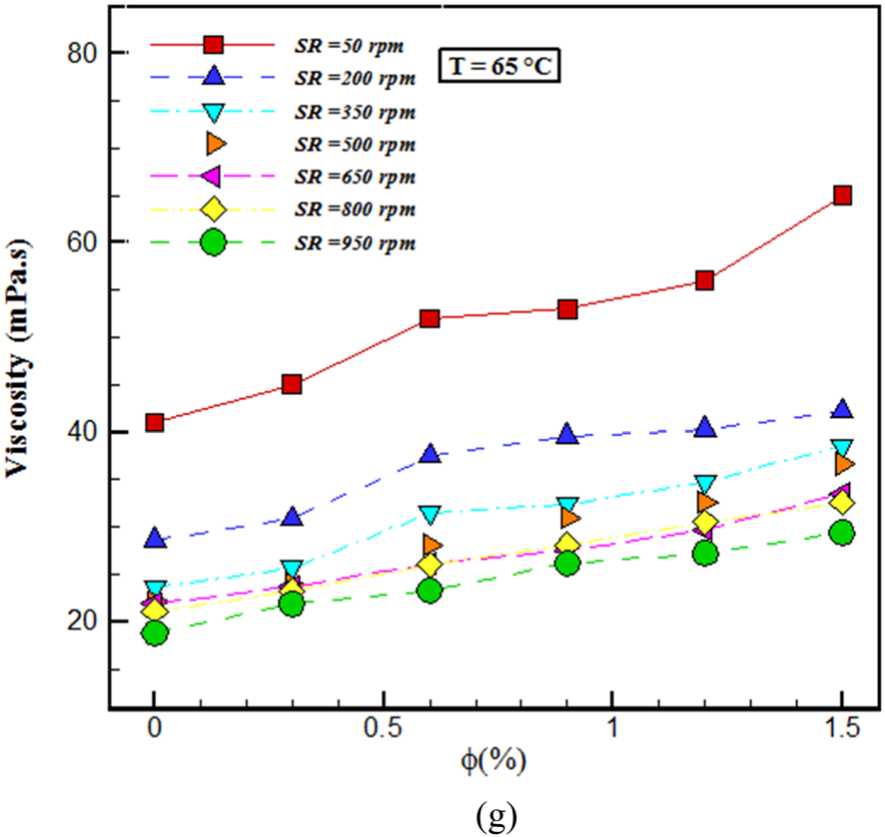
Viscosity changes with $\varphi$ in different conditions.

Figure 6 show the variations in $\mu$ in terms of $\varphi$ and SR. It can be seen that at different temperatures, with the enhancement in $\varphi$, the $\mu$ decreases, and showing the direct relationship between $\varphi$ and viscosity. The volume fraction strengthens the IMF and the movement of molecules declines. For example, at SR= 50 rpm, with a change in $\varphi$ from 0 to 1.5% at *T*= 65, 45, and 15 °C, about 58, 62, and 42% increase in fluid viscosity was seen, respectively. Also, the highest enhancement in the $\mu$ occurred at *T* =35 °C, SR = 50rpm and $\varphi$ =1.5, which is about 76.88%.

### Uncertainty

Standard solution for calibrating the viscometer was deionized water. There is an accuracy of 0.1°C when measuring temperature (5 to 65 °C). Viscosity is measured with a viscometer that is accurate to within 2.0%. Based on equation (4)^56^, the maximum uncertainty (${A}_{\mu })$ is 2.8% at SR=200RPM.

4 $${A}_{\mu }=\sqrt{{\left(\frac{\delta T}{T}\right)}^{2}+{\left(\frac{\delta {\mu }_{nf}}{{\mu }_{nf}}\right)}^{2}}.$$

The uncertainty of wear rate ($${A}_{wear rate})$$ is computed as follow as:

5 $${A}_{wear\, rate}=\sqrt{{\left(\frac{\delta \Delta m}{\Delta m}\right)}^{2}+{\left(\frac{\delta \rho }{\rho }\right)}^{2}+{\left(\frac{\delta l}{l}\right)}^{2}{+\left(\frac{\delta F}{F}\right)}^{2}},$$

where the accuracy for measuring $\Delta m$, $l$, $\rho$ and $F$ are 2.171, 1,0.003, and 0.2%, respectively. The maximum value of ${A}_{wear rate}$ is 2.39%.

### Comparison of relative viscosity to established models

**Figure 7:**
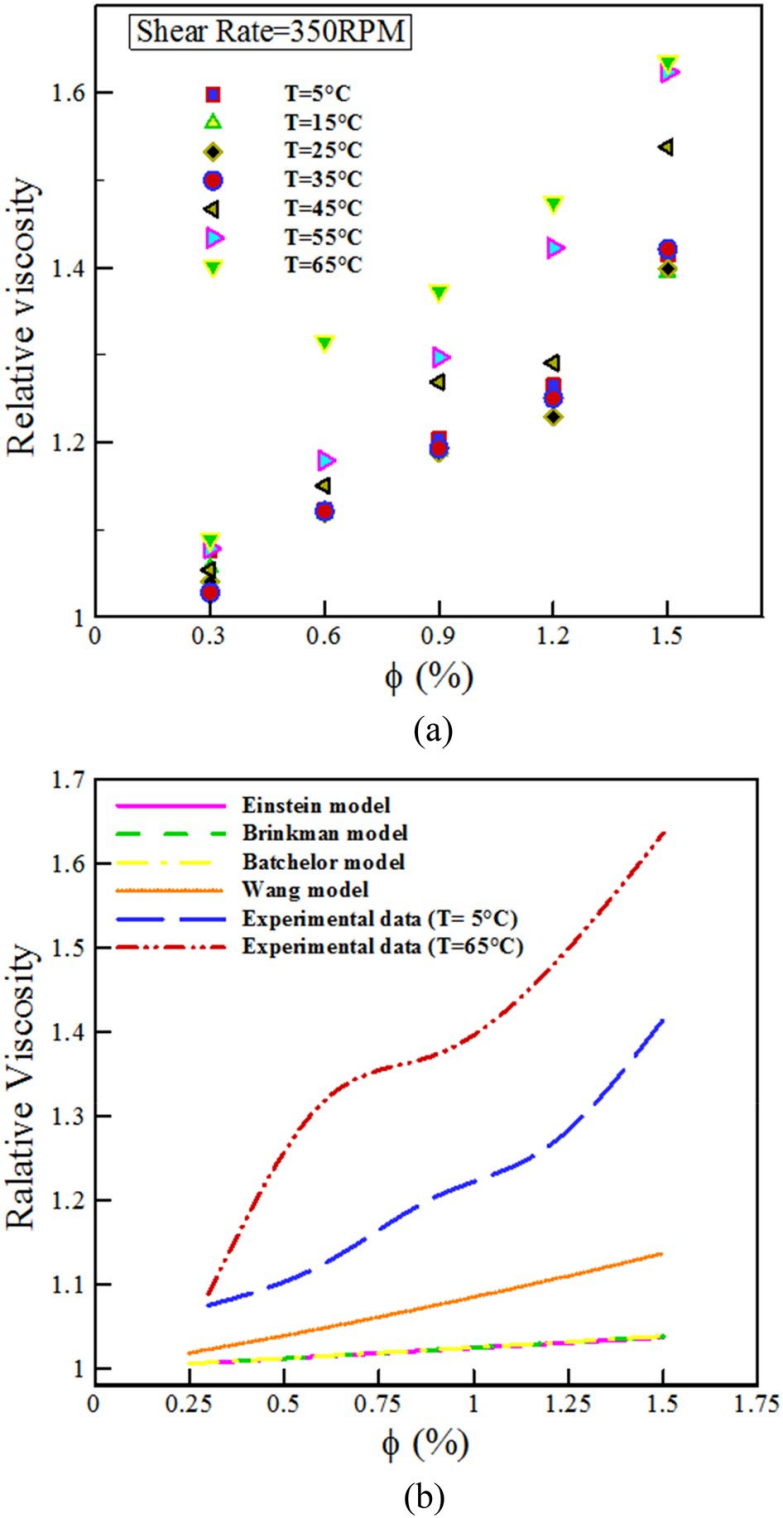
(**a**) Relative viscosity changes in terms of $\varphi$ and T and (**b**) Evaluation of results with previous models.

Figure 7a explains the relative $\mu$ changes with $\varphi$ for different *T* at SR= 350 rpm. ${\mu }_{nf}> {\mu }_{bf}$ in all $\varphi$. Sepehrnia et al.^39,40^ in their research showed that the values of the relative $\mu$ of the THNF is greater than the unit value. The results in Fig. 7 show that at low $\varphi$, the relative viscosity values are close to each other at different temperatures, but as the $\varphi$ increases, the relative viscosity values at the minimum and maximum temperatures differ greatly, and it gets its highest value at $\varphi$=1.5%. The results show that the greatest rise in $\mu$ compared to the BF at a constant temperature is 63.56%, which corresponds to the NF with $\varphi$= 1.5% and *T*= 65 °C; the lowest rise in $\mu$ compared to the primary fluid is 56.7%, which relates to the NF with $\varphi$ = 0.25% and *T*= 5 °C.

Various models have been presented to calculate relative viscosity, which will be introduced below. To compute the relative $\mu$, Einstein presented the subsequent relationship^57^.

6 $$\frac{{\mu_{nf} }}{{\mu_{bf} }} = 1 + 2.5\phi ,$$

where *nf* and *bf* are specified the NF and base fluid. Brinkman^58^ developed the model presented by Einstein as follows:

7 $$\frac{{\mu_{nf} }}{{\mu_{bf} }} = \frac{1}{{(1 - \phi )^{2.5} }}.$$

Batchelor^59^ based on the assumption of spherical shape for NPs and its uniform distribution in the fluid and considering the Brownian motion of NPs presented the Eq. (8) for $\frac{{\mu_{nf} }}{{\mu_{bf} }}$:

8 $$\frac{{\mu_{nf} }}{{\mu_{bf} }} = 1 + 2.5\phi + 6.2\phi^{2} .$$

Wang^60^ presented the Eq. (9) to calculate the $\frac{{\mu_{nf} }}{{\mu_{bf} }}$:

9 $$\frac{{\mu_{nf} }}{{\mu_{bf} }} = 1 + 7.3\phi + 123\phi^{2} .$$

Figure 7b compares the relative viscosity of the outcomes of the current study at T= 5 and 65 °C and SR = 350 rpm with Einstein^57^, Brinkman^58^, Batchelor^59^ and Wang^60^ models. As it is known, the models of Einstein, Brinkman, Batchelor and Wang show a linear behavior, while the results of the present study have a non-linear behavior at T = 5 and 65 °C. According to Fig. 7b, the previous common models do not have the power to forecast the behavior of the THNF of the present study. Therefore, novel modeling for the $\mu$ of this NF should be done.

## Results of tribological tests

**Figure 8:**
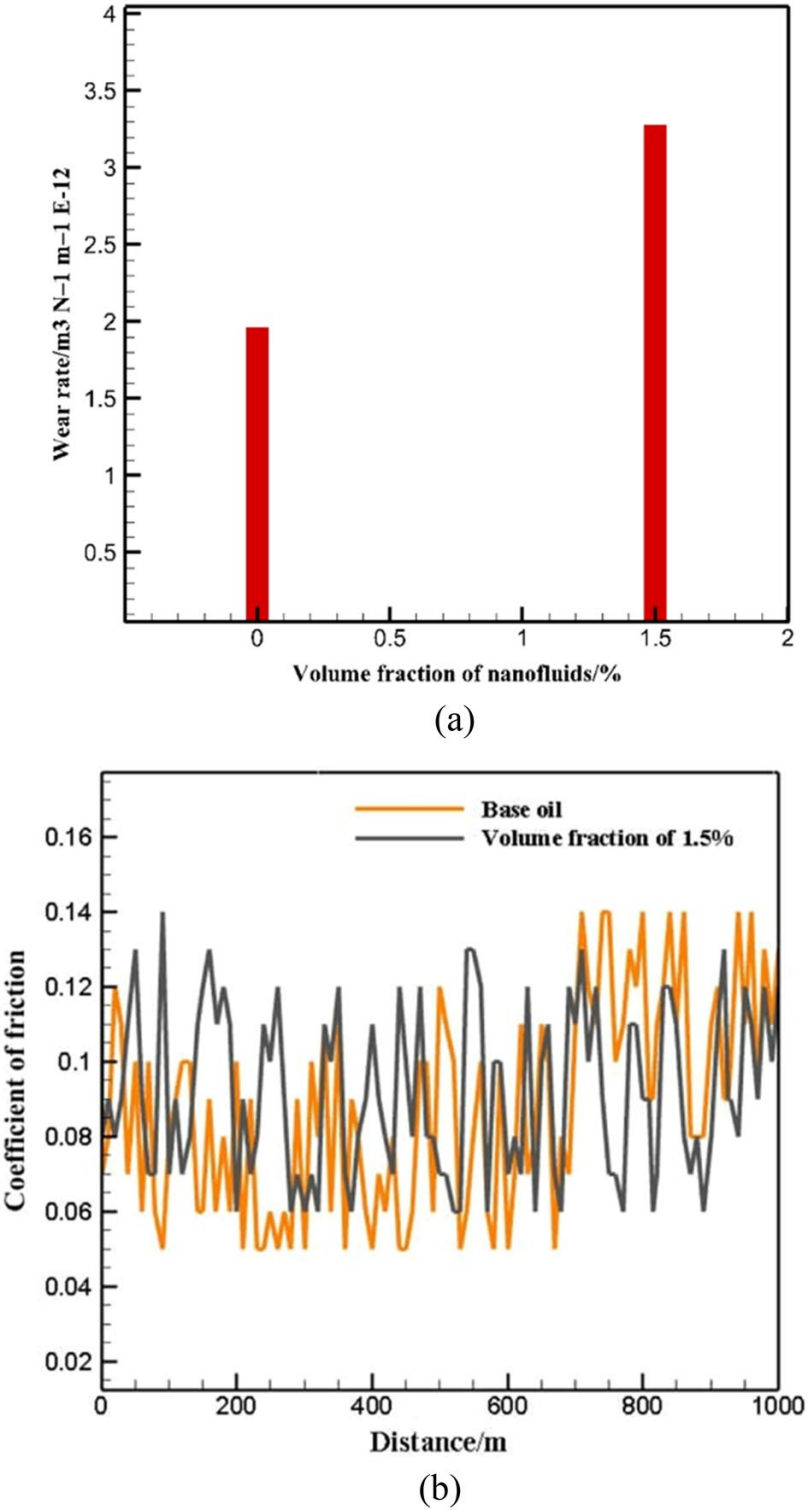
Changes in (**a**) wear rate with $\varphi$ and (**b**) FC with distance.

Figure 8 shows the change in wear rate according to volume fraction and distance. The graph results show that by adding NPs to the primary fluid, the wear rate has increased by 35%. In this test, the engine speed is 100 rpm, the average applied force is 195 N and $\varphi$ = 1.5%. Table 3 shows the sample mass reduction in the tests performed for 0 and 1.5% mass. In the current research, it has been observed in the THNF that the friction coefficient has increased by about 35% at $\varphi$= 1.5%. In many examinations, it has been shown that factors such as chemical composition, size, volume fraction, and shape of NPs affect the lubrication performance. In studies, it has been shown that in a specific $\varphi$, the minimum friction coefficient for nano-lubricant is observed, for example, Mosleh et al.^43^ displayed that mounting the $\varphi$ of molybdenum disulfide NPS to oil first lessens and then rises the FC, so that in NF with a $\varphi$= 0.5%, the FC is minimized and with an enhancement in the $\varphi$, the FC increases. Jiao et al.^61^ displayed that enhancing the $\varphi$ in Al_2_O_3_-SiO_2_-oil BHNF from 0 to 0.5% decreases the FC and from 0.5 to 1% increases the FC. Also, base fluid can play a significant character in NF's tribological behavior; In this regard, Wu et al.^42^ showed that adding diamond NPs to SAE30 LB51163-11 oil reduces the friction coefficient, while adding it to SAE30 LB51153 oil increases the friction coefficient. The existence of liquid oil significantly lessens the phenomenon of aggregation, but does not disregard it. So, added NPs in improved oils can aid reduce the accumulation of wear particles at the interface and improve the total wear rate. However, if the $\varphi$ surpasses the permissible limit, it may impair the efficiency of the fluid lubricant in eliminating trapped wear deposits. Also, in this study, the amount of FC changes up to 100m for both base oil and oil containing 1.5% of NPs has been investigated. The average value of FC for NF with $\varphi$ of 0% and 1.5% is 0.088 and 0.092, respectively. The FC with $\varphi =$ 1.5% is greater than the primary fluid. The wear rate for the desired NF at $\varphi =$ 1.5% has increased by almost 68.4% compared to the primary fluid. As mentioned in the reference, in high $\varphi$, there is a possibility of accumulation of particles and increase of FC and wear rate. Perhaps the reason of this behavior can be attached to the $\varphi$ and the percentage of NPs composition in the THNF, because the presence of spherical NPs can be effective in reducing the wear rate. In the current survey, the presence of NPs in the $\varphi =$ 1.5% in this psychoanalyzer could not cause the rolling mechanism and reduce the wear rate.

**Table 3:** Mass reduction of the samples under the wear test at distances of 1000 m.

Volume fraction (%)	The weight of the prototype	Sample weight after a distance of 1000 m	The amount of sample weight loss (1000 m)
0	92.025	92.020	0.005
1.5	91.983	91.980	0.003

The statistical analysis of the data is shown in Table 4. An indicator of relative variability is the coefficient of variation (CV). In other words, it is the ratio between the standard deviation and the mean.

**Table 4:** Statistical analysis of the data.

	Input data			Output
	T (°C)	SR (rpm)	Volume fraction	Viscosity (mPa.s)
Min	5.0000	50.0000	0.0000	18.8000
Max	65.0000	950.0000	1.5000	600.0000
Mean	33.8235	500.0000	0.7500	137.2467
Standard deviation	20.6540	324.0370	0.5612	127.0940
Coefficient of variation	0.6106	0.6481	0.7483	0.9260

## Soft computing model

### Neural network (NN)

Artificial neural networks (ANN) consist of a great number of highly interrelated processing elements called nerve cell that work collected to solve a problem. The classification of NN is a network of layers, usually the layer to which the input information is given is called the input layer and the layer from which the output data is received is named the output layer, and the other layers among these two layers (if any) are named hidden layers. The direction of movement of the signals is always from the side of the input layer to the output layer. To classify a test sample, the weight of the words is determined for the input units, and the initiation of these components is done through different forward layers in the ANN, and the value of the output unit is a result in decision-making. Categories are determined. ANN are computational systems stimulated by the organic NN that make up animal brains. The meaning of learning in NN is to regulate the weights and biases of the network. Meta-heuristic algorithms can determine the weights of edges and biases in NNs. Therefore, in this study, the weight of biases and edges is determined using the genetic algorithm. In this part, using soft calculations, prediction of $\mu$ is discussed using available laboratory data, 289 data. The input variables are T, VF and SR. In preparing the data for teaching this point, the maximum and minimum of all three variables were included in the training set. In this research, feed-forward neural networks with back-propagation architecture were used. These types of networks are very efficient in modeling the non-linear relationship of variables. Since the goal is to predict the viscosity of NFs, the output of the neural network was determined, the relative thermal conductivity of NFs, and the parameters affecting it, i.e. the volume fraction of NP, temperature, SR were selected as the inputs of the neural network. During the execution of the training algorithm, the input data were entered as input to the input layer of the neural network, and the difference between the network output (outputs) and the desired output (targets) was used as a criterion for correcting the weights and biases. It is worth mentioning that at the beginning of all the weights and biases are assigned randomly.

The training of the network is carried out by the Levenberg–Marquardt algorithm optimization algorithm, by which the optimal values of the weights and biases are found. One of the advantages of this algorithm is its speed and accuracy in the neural network training process. Transfer functions (purelin-tansig) with 18 neurons produced the least error. Therefore, this structure was used to design the ANN model. In the current research, the data are divided into 85% for training and 15% for testing. MATLAB software is used in this study^62^.

### Adaptive network-based fuzzy inference system (ANFIS)

ANFIS is a mixture of a fuzzy system and an artificial NN in such a way that it includes the advantages of both. This system is useful for solving non-linear and complex problems. ANFIS can establish and infer the non-linear connection between inputs and outputs with the help of linguistic concepts. Compared to ANN, ANFIS is trained faster and more accurately due to the adjustable parameters of the fuzzy system. Sugeno's neural-fuzzy inference system is used in this study. The Sugeno system performs better in calculations and has an actual output. In Sugeno system, the antecedent part of the benefits is fuzzy, but the result part is non-fuzzy and a linear combination of input variables. In ANFIS, it is necessary to specify the type of membership function and its frequency in the first layer. In the current research, the data are divided into 85% for training and 15% for testing. MATLAB software is used in this study. Sub-clustering with influence Radius, maximum of Epochs number and initial step size: 0.3, 200, and 0.01 are used^62,63^.

### Machine learning-Gaussian process regression (ML-GPR)

In general, ML is classified into two groups and acting different procedures depends on the data: Supervised and Unsupervised. GPR is a non-parametric Bayesian method to regression that makes waves in the field of ML.GPR models do not need any validation, and GPR can realize the prediction data matching to the test data^64,65^. Cross-validation can be used to detect overfitting in the model. 10% of data is for cross-validation. The molding was performed in MATLAB software. In this software, GPR is a set of Gaussian process regression models trained on cross-validated folds. Base on minimum MSE, hyper-parameters are: Sigma = 0.268; kernel function = nonisotropic matren 5/2; Basis function = Zero.

In the present study, R-square and RSME for the desired parameter(y) are used to compare easier models:

10 $$RSME=\sqrt{\frac{\sum_{i=1}^{N}{\left({y}_{exact,i}-{y}_{estimted,i}\right)}^{2}}{N}},$$

11 $$R-square=1-\sum_{i=1}^{N}\frac{{\left({y}_{exact,i}-{y}_{estimted,i}\right)}^{2}}{{y}_{exact,i}}.$$

**Figure 9:**
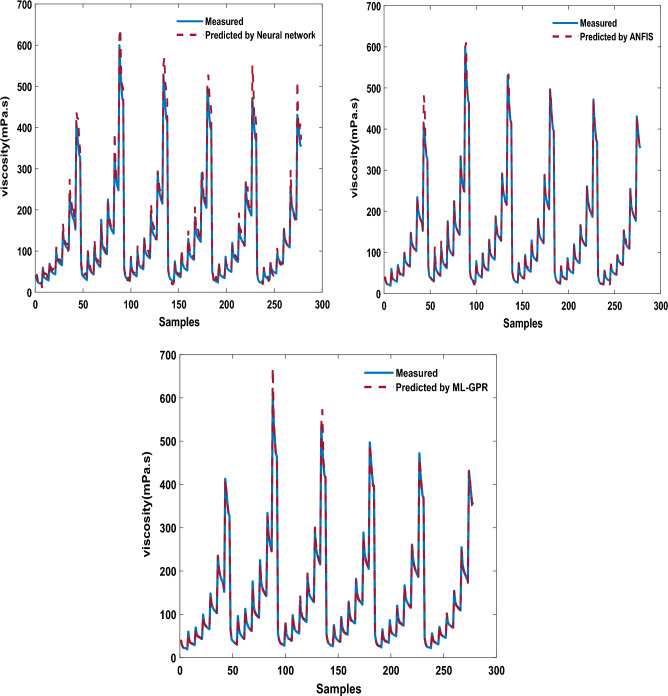
Estimation of viscosity using the proposed models.

**Figure 10:**
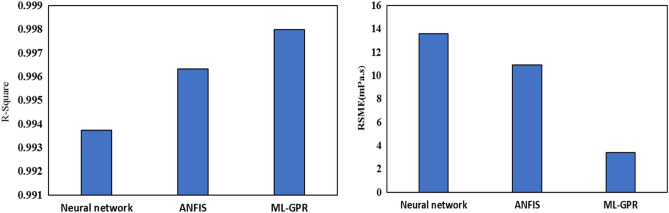
R-square and RSME for the proposed models.

*N* is the number of experimental data. Modeling of all three proposed methods has been done in MATLAB software. 75% of data were used for training, 10% for cross-validation and the remained ones applied for testing. Using the proposed models, the $\mu$ of the THNF has been estimated, and the outcomes are revealed in Fig. 9. The outcomes specify the high accuracy of all three mentioned models. For a better comparison, the value of *R-square* and *RSME* for all three models is presented in Fig. 10. All three models have a high ability to estimate fluid viscosity based on T, $\varphi$ and SR. Also, the lowest error value of RSME is related to ML-GPR. The results of present research can be used in a wide range of engineering systems such as heat exchangers^66^, enclosures^67^, solar collectors^68^, heat sinks^69^, automotive radiators^70^, porous medias^71^, nano-lubricants^72^, cooling of equipments^73^, heat pipes^74^, and microchannels^75^.

## Conclusion

In this exploration, the $\mu$ and wear of THNF of oil (5W30)-GO-SA-MWCNTs in the *T*= 5–65 °C, $\varphi =0-1.5\%$ and the SR = 50–950 rpm was investigated. The findings of this research are given below:

1. Changes in shear stress with SR specify that the NF has a NNB of a pseudo-plastic type (power index less than one). By augmenting the SR and falling the *T*, the $\mu$ rises. With the enhancement in the $\varphi$, the viscosity increases about 38-72%. At a constant temperature, shear stress increases with augmenting $\varphi$, and at a constant $\varphi$, shear stress lessens with enhancing *T*. The highest $\frac{{\mu_{nf} }}{{\mu_{bf} }}$ occurs at T= 55°C and $\varphi =$ 1.5%, which shows that the NF has increased viscosity by 72% compared to the primary fluid. In all $\varphi$, dynamic viscosity decreases with augmenting SR.
2. The wear test of the pin on the disc is used to inspect the wear of NF. By adding NPs with $\varphi$ = 1.5% to the base fluid, the wear rate has increased by 68%. In the base oil, with growing distance, the FC has reduced by 4.5% compared to the oil containing NPs.
3. Using the obtained laboratory data and NN models, ANFIS and GPR ML, the viscosity of THNF has been estimated. All three models predicted the viscosity well. The lowest RSME value belongs to the GPR ML model.

## Data Availability

All data analyzed during this study are included in this published article.

## List of symbols

*T*: Temperature ($$^\circ{\rm C} )$$
$$m$$: Mass (kg)

## Greek

$\varphi$: Volume fraction of nanopowders
$\tau$: Shear stress (Pa)
$$\rho$$: Density (kg.m^–3^)
$\upmu$: Dynamic viscosity (Pa.s)

## Subscripts

bf: Base fluid
nf: Nanofluid

## Abbreviations

ANFIS: Adaptive neuro-fuzzy inference system
ANN: Artificial neural networks
BHNF: Binary hybrid nanofluid
GPR: Gaussian process regression
HT: Heat transfer
IMF: Intermolecular force
ML: Machine learning
NN: Neural network
NF: Nanofluid
NPs: Nanopowders
NNB: Non-Newtonian behavior
SR: Shear rate
THNF: Ternary hybrid nanofluid
